# Feasibility of a Standardised Mid‐Trimester Ultrasound Protocol: A National Multicenter Study

**DOI:** 10.1111/1471-0528.18102

**Published:** 2025-02-13

**Authors:** Thierry Bultez, Laurent Julien Salomon, Houman Mahallati, Nicolas Fries, the CNEOFT2 Study Group, Sara Amat, Bettina Bedel, Guillaume Benoist, Roger Bessis, Amaury Boleis, Gihad Chalouhi, Sophie Delahaye, Benjamin Deloison, Ferdinand Dhombres, Luc Durin, Xavier Favre, Anaig Flandrin, Laurence Gitz, Pierre Godard, Antoine Lafouge, Jean Marc Levaillant, Mona Massoud, Raphaelle Mangione, Pierre‐Antoine Migeon, Deborah Perrot, Franck Perrotin, Thibaud Quibel, Pascale Sonigo, Pierre Uzan, Nicolas Sananes

**Affiliations:** ^1^ Collège Français d'Echographie Foetale, CFEF Paris France; ^2^ Assistance Publique‐Hôpitaux de Paris, Groupe Hospitalier Pitié‐Salpêtrière, Sorbonne Universités Paris France; ^3^ Assistance Publique‐Hôpitaux de Paris, Hôpital Necker‐Enfants Malades Paris France; ^4^ Department of Radiology, Cumming School of Medicine University of Calgary Calgary Canada

**Keywords:** audit, prenatal, protocol, quality assurance, screening, sonography, standard, ultrasound

## Abstract

**Objective:**

Evaluate the feasibility and quality of a national standardised mid‐trimester ultrasound protocol using a consensus‐based quality assessment (QA) scoring system.

**Design:**

Multicenter prospective observational ‘FLASH’ study.

**Setting:**

(i) Assessing the feasibility of a standardised protocol of 24 views at the mid‐trimester scan, with 21 recommended and 3 additional views, in routine practice.

(ii) Assessing the quality of these images by evaluating the presence of conformity criteria.

(iii) Analysing the reliability between self‐assessment and peer‐assessment of the images.

**Population:**

A total of 440 mid‐trimester scans.

**Methods:**

A consensus‐based QA scoring system comprising 73 conformity criteria was established with 28 experts using a 3‐round Delphi method. Secondly, we asked operators to record 5 consecutive routine mid‐trimester scans. Images were analysed by the sonographer themselves and by a qualified expert according to the scoring system. The frequency of recorded images was calculated for each of the views. Factors associated with missing images per scan were evaluated. The robustness of conformity criteria was assessed by reliability between self‐evaluation and peer‐evaluation.

**Main outcome measures:**

Based on 9849 images, we observed feasibility of the 21 recommended standardised views for mid‐trimester scan ranging from 88.5% to 100%.

**Results:**

Most conformity criteria (64/73, 88%) were met in over 90% of cases. Gwet's AC1 correlation between expert evaluation (peer‐evaluation) and participant evaluation (self‐evaluation) was greater than 0.80 for 70/73 (96%) criteria.

**Conclusion:**

This large‐scale 2‐month ‘flash’ observational study demonstrates the feasibility and quality of a national standardised mid‐trimester ultrasound protocol using a consensus‐based QA scoring system.

## Abstract 2

**Linked article:** This article is commented on by Fantasia and Volpe pp. 1074–1075 in this issue. To view this article visit https://doi.org/10.1111/1471‐0528.18137.

## 1 Introduction

A significant proportion of morphological anomalies can be detected by a detailed ultrasound scan of foetal anatomy at 20 + 0 to 24 + 6 weeks, also known as the mid‐trimester scan [1, 2, 3]. Several studies have shown that the adoption of a systematic scan with a standardised protocol is associated with improved detection rate of anomalies [4, 5, 6]. The quality of stored images reflects the overall quality of foetal anatomical assessment, and regular audit procedures are recognised as a mean of maintaining and improving practice quality [7, 8].

Various scientific organisations have established protocols for the mid‐trimester ultrasound scan, including the International Society of Ultrasound in Obstetrics and Gynaecology (ISUOG) [2, 9] in 2013 and 2023.

**FIGURE 1:**
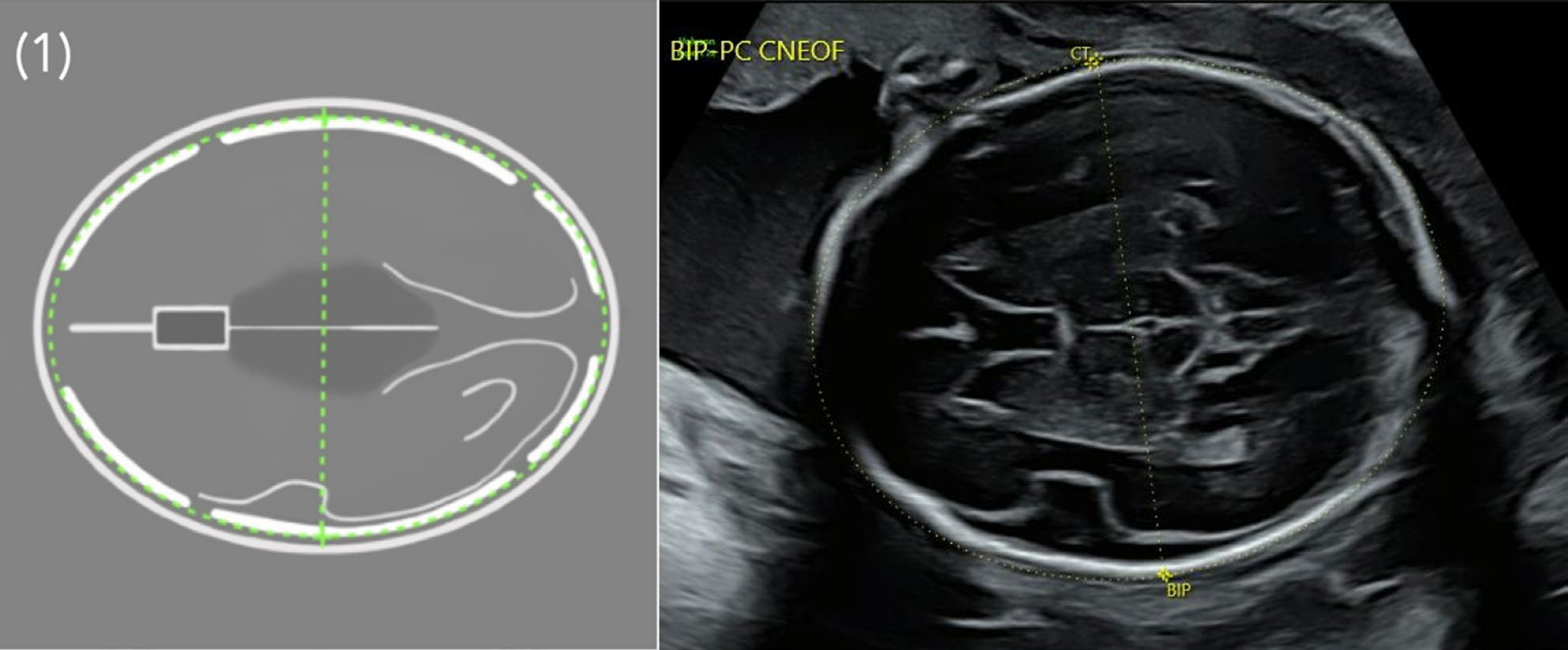
Standardised axial view of the cephalic pole illustrated in the form of a ‘silhouette’ and the corresponding ultrasound image at 24 weeks. In this case, the ‘silhouette’ illustrates the cavum septi pellucidi (CSP) on the axial transthalamic view. The CSP is one of the conformity criteria of the consensus‐based QA scoring system for this standardised view.

In France commencing in 2005, the Comité Technique d'Echographie (CTE) [10] and then the Conférence Nationale d'Echographie Obstétricale et Fœtale (CNEOF) [11] have issued recommendations on ultrasound protocols for the principle US scans during pregnancy in each of the three trimesters. These recommendations have been accepted as the professional standard to be adhered to and are referred to in cases of malpractice litigation. The CNEOF 2022 mid‐trimester protocol includes a total of 26 standardised views, 23 recommended and 3 additional views. As the views of limbs are often grouped into one image, this results in a total of 24 views, of which 21 are recommended. If the recommended views are not obtained, the additional views can be produced to complement the recommended views that were obtained. The choice of these standardised views was justified by their a priori feasibility in current practice, their ability to illustrate items detailed in the text of the ultrasound report, and to include anatomical structures affected by the most common and severe foetal morphological anomalies. These standardised views are illustrated in the form of ‘silhouettes’ and are deliberately schematic to avoid subjective interpretation of a ‘typical’ ultrasound image (Figure 1).

The aims of this ‘flash’ study were (i) to evaluate this new standardised protocol by assessing the feasibility of performing the selected mid‐trimester views in routine practice, (ii) to assess the quality of these images by evaluating the presence of anatomical landmarks and conformity criteria and (iii) to analyse the reliability between self‐assessment and peer‐assessment of the images in order to prepare for the quality‐control programme that should soon accompany the national implementation of these new recommendations in France.

## 2 Methods

### 2.1 Developing a Consensus‐Based Quality Assessment (QA) Scoring System

Prior to data collection, the 26 standardised views from the CNEOF 20 + 0 to 24 + 6 week ultrasound scan protocol were submitted to 28 experts *(operators performing > 300 mid‐trimester scan year for the past 10 years and working in reference hospitals)* to establish a consensus‐based quality assessment (QA) scoring system based on ultrasound conformity criteria using a 3‐round Delphi method. All experts were free to recommend additional experts with the required expertise. There was no restriction on the invitation of experts from the same institution or country. An invitation email was sent to the potential participants. Those who agreed to participate received an email explaining the Delphi process, a questionnaire, and instructions on how to complete the questionnaire. The criteria of this table were defined as conformity criteria, as it is sometimes difficult to distinguish between quality and normality criteria, that is, the usual appearance of the structure (*as an example, the non‐visibility of cavum septi pellucidi on the transthalamic axial view of foetal head may correspond to an inadequate view or to a true absence as can be seen as an indirect sign of agenesis of corpus callosum*).

The aims of these questionnaire were: [1] to explore agreement between the experts on the proposed conformity criteria to identify criteria that might need a revised definition because of poor agreement; [2] to collect suggestions for revised definitions; and [3] reach consensus on suggested revised definitions. The questionnaires included an Excel sheet with the list of the 145 of possible conformity criteria.

Experts were asked to suggest conformity criteria for analysing the 26 views: 23 recommended and 3 additional for singletons. As the views of both upper and lower limbs could be grouped together on a double image, 24 views were studied, 21 recommended and 3 additional. This resulted in a list of 145 possible conformity criteria, which were then resubmitted to the same 28 experts.

A 1–9 Likert numerical scale was placed against each criterion to agree on their appropriateness [12]:

- A value of 1 means that the expert considers the proposed criteria as ‘totally inappropriate’ (or not indicated, or not acceptable);
- A value of 9 means that the expert judges proposed criteria to be ‘totally appropriate’ (or indicated, or acceptable);
- Values 2 to 8 represent intermediate scenarios.
- The value ‘5’ corresponds to indecision.

Each expert was asked to return the completed questionnaires by email to N.F. and remained blinded to the other experts' answers.

After the first round, we eliminated those criteria with median value ≤ 3.5 and retained those criteria that had obtained median value ≥ 7. Those between these two values were subject to a 2nd round, at the end of which we retained only those criteria that received a median value ≥ 7. Consequently, each criterion has the same weight.

All the experts participated in all three rounds.

Finally, we established a consensus‐based QA scoring system comprising 73 criteria out of the 145 initially proposed with the number of criteria per view ranging from 1 to 8. (Table S1).

### 2.2 Data Collection

This multicenter observational ‘flash’ study [13, 14, 15] was conducted from May, 2023 to July 14, 2023. We asked operators registered with the French college of ultrasound (CFEF) to record 5 consecutive patients on whom they were carrying out routine mid‐trimester ultrasound screening on singletons, between 20 + 0 and 24 + 6 weeks, using a secure digital interface. Before starting the study, the operator was given access to the quality assessment (QA) scoring system and a short video explaining how to record the data.

CFEF members could be either in the diagnostic membership group, including those members acting as reference physicians who perform more than 1000 obstetrical ultrasounds per year, or in the screening membership group in which they perform at least 300 routine obstetrical ultrasounds per year. The latter group includes both physicians and midwives.

Inclusion criteria were patients undergoing their routine mid‐trimester ultrasound scan. Non‐inclusion criteria were pregnancy with a gestational age < 20 + 0 or > 24 + 6 weeks. The patient was asked for verbal or written consent to participate in this non‐interventional study. This study was approved by the local ethics board under CEROG 2023‐OBS‐0405 [16].

For each case, the sonographer filled in the demographic data that included age, weight, height, parity, gestational age based on first trimester measurement of CRL, duration of the scan, history of caesarean delivery, history of laparotomy, presence of fibroids, poor technical factors, foetal position, placental position, use of transvaginal ultrasound and use of a software‐supported checklist during the scan. The sonographer was then asked to send the anonymized images corresponding to the 21 recommended views and the 3 additional views. For this study, operators were asked to perform the additional 3 views routinely.

All recorded images were then analysed by the sonographer themselves and also by a qualified expert (NF) according to the criteria defined in the scoring system. Both the sonographer and the expert were blinded to the scores until the 2 evaluations had been finalised.

The evaluation of each criterion was binary: depending on whether the criterion was met. A view was considered not feasible if it had not been recorded by the sonographer. The frequency of recorded images was calculated for each of the views. Factors associated with missing images or specific anatomic planes per scan were evaluated.

The robustness of conformity criteria was assessed by reliability between self‐evaluation and peer‐evaluation.

### 2.3 Statistical Analysis

All statistics were calculated using RStudio software (R Core Team) (2016). R: A language and environment for statistical computing. R Foundation for Statistical Computing, Vienna, Austria. https://www.R‐project.org/ [17]. Wilcoxon nonparametric and Spearman's correlation tests were used for continuous variables. All ‘*p*’ values were reported two‐sided; a p test value < 0.05 was considered statistically significant. Reliability analysis between hetero‐evaluation and self‐evaluation was performed using Gwet's AC1 statistic, more appropriate due to a high prevalence of view conformity criteria [18]. AC1 values < 0.6, between 0.6 and 0.8 and > 0.8 were taken to represent poor, moderate and good reliability, respectively.

Finally, a view was considered as very highly compliant if all conformity criteria per view were present at over 95% and if the AC1 for each criterion was > 0.8, as highly compliant if at least one criterion was between 90% and 95% and an AC1 > 0.8 and as moderately compliant if at least one criterion was < 90% or an AC1 < 0.8.

## 3 Results

### 3.1 Population

From 01/05/2023 to 14/07/2023, 88 CFEF members took part in this study: 19 members from the diagnostic membership group (22%) registered 95 cases, 69 members from the screening membership group (78%) registered 345 cases. Operators were uniformly distributed across France, in public and private hospitals, and in private practice.

We collected a total of 440 mid‐trimester scans.

The characteristics of the population are summarised in Table 1.

**TABLE 1:** General characteristics of the population.

		Population (*n* = 440)
Age (years) median (IC95%), *n* = 440		31 (30–32)
Gestational age (weeks) median (IC95%), *n* = 440		22.5 (22.4–22.7)
Nulliparous, *n* (%)		197/436 (44.7)
BMI (kg/m^2^), median (IC95%), *n* = 440		24.6 (24.2–25.1)
BMI > 30 kg/m^2^, *n* (%)		59/413 (14.3)
Previous caesarean section, *n* (%)		46/437 (10.5)
Previous laparotomy, *n* (%)		2/439 (0.5)
Presence of fibroids, *n* (%)		5/435 (1.1)
Technically challenging conditions, *n* (%)		131/428 (30.6)
Foetal position, *n* (%)	Cephalic	232/431 (53.8)
	Breech and transverse	199/431 (46.2)
Spine position, *n* (%)	Anterior or posterior	185/431 (66.6)
	Lateral	144/431 (33.4)
Anterior placenta, *n* (%)		207/431 (48.0)
Examinations performed by members of screening group, *n* (%)		345/440 (78.4)
Examination time (min), median (IC95%), *n* = 440		28 (27–30)
Use of vaginal route, *n* (%)		75/435 (17.2)
Use of a software‐supported checklist, *n* (%)		92/430 (21.4)

### 3.2 Feasibility

The CNEOF 2022 recommends 24 views (21 recommended +3 additional) during the performance of a screening mid‐trimester ultrasound. A total of 9849 images were recorded out of the 10 560 (440 × 24) expected images (93.3%), 9028/9240 (97.8%) recommended images and 821/1320 (62.2%) additional images. The frequency of recorded images according to recommended and additional views is detailed in Table 2.

**TABLE 2:** Frequency of recorded images according to recommended and additional views.

	Number	%
Recommended standardised Views		
Axial view of the cephalic pole	439	99.8
Trans‐cerebellar axial oblique cephalic view	438	99.6
Midsagittal cephalic view	428	97.4
Coronal view of the face centered on the nose and mouth	433	98.5
Axial view of the orbits	431	98.0
Midsagittal profile view	434	98.7
Four‐chamber view	439	99.8
Left outflow tract view	437	99.3
Three vessels and trachea view	432	98.3
Bifurcation of the pulmonary artery view	420	95.7
Axial view of the abdomen through the portal sinus	438	99.6
Axial view of the abdomen through the gallbladder	429	97.6
Axial view of the kidneys	432	98.3
Midsagittal view of the lumbosacral spine	430	97.8
Left parasagittal view of thorax and abdomen	436	99.1
Oblique view of the pelvis through the umbilical arteries and the bladder	430	97.8
Longitudinal view of the femur	440	100
Longitudinal view of the 2 femurs	387	88.5
Lower limbs view	427	97.2
Upper limbs view	423	96.3
Midsagittal view of the uterus through the cervix	425	96.7
Additional standardised Views		
Transverse view of the abdomen at insertion level	340	78.3
View of the right sided cardiac cavities surrounding the aorta	251	58.9
Coronal view of the cephalic pole	230	54.3
Total	9849	93.3

Feasibility was > 95% for 20 of the 21 recommended views. Only the view of the two femurs had a feasibility < 95% (88.5%). The 3 additional views were performed in 54.3% to 78.3% of cases.

**FIGURE 2:**
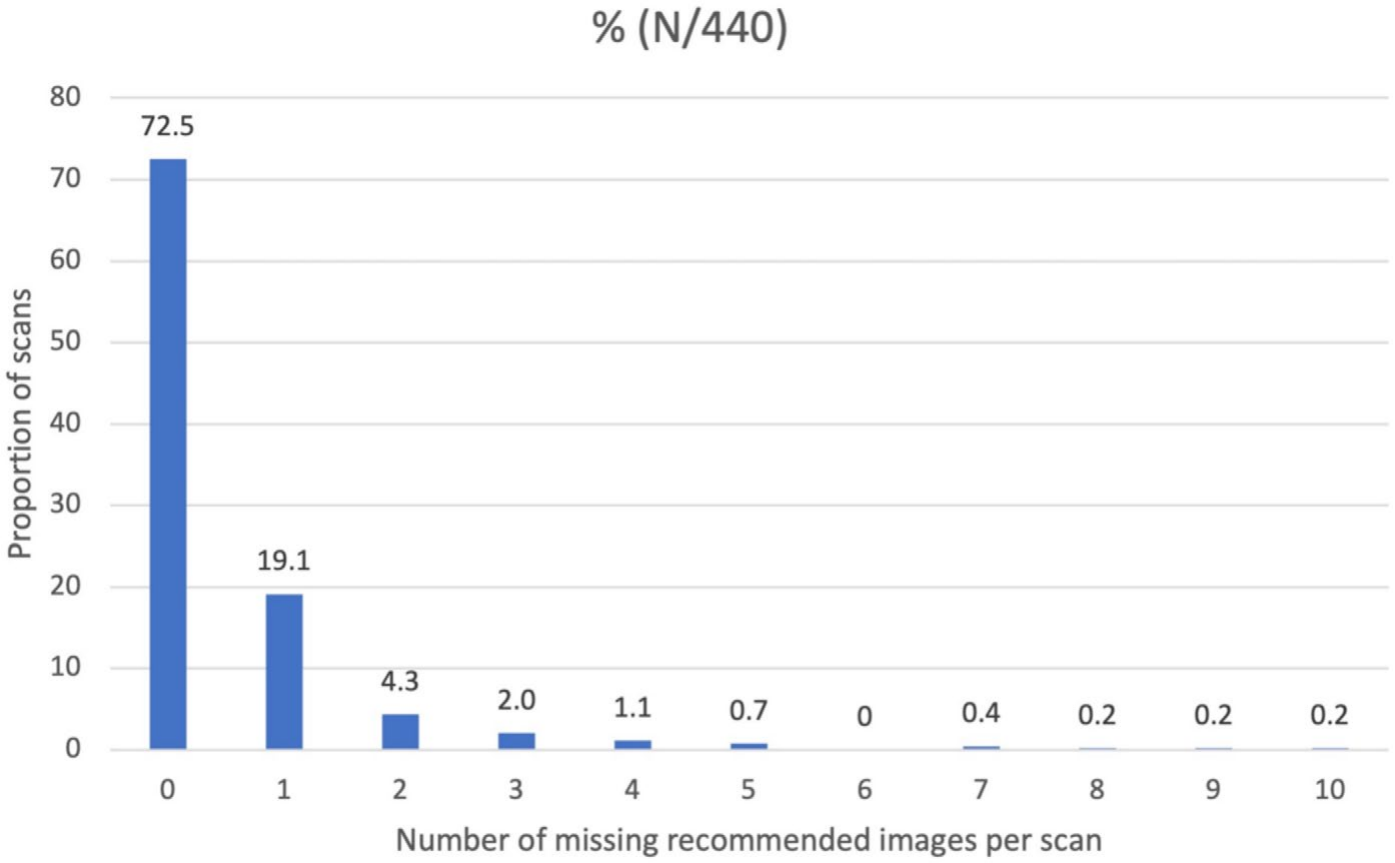
Proportion of scans as a function of the number of missing recommended images per scan (24 recommended views).

The relative portion of scans according to the number of missing recommended images per scan is detailed in Figure 2. Of the 440 scans, 319 (72.5%) had none of the 21 recommended views missing. Although there was no recommended time allocated for the scan, the median scanning time was 28 min (IC95% 27–30). A shorter scan time was significantly associated with a higher number of missing images (*p* = 0.016).

The median of missing images per exam was also significantly associated with a BMI > 30 kg/m2 (0.4 vs. 0.7, *p* = 0.04). Members of the screening group did not have statistically a higher median of missing images than members of the diagnostic group (0.5 vs. 0.4, *p* = 0.35). Age, parity, previous caesarean, previous laparotomy, presence of fibroids, gestational age, placental position, technically challenging conditions, use of transvaginal ultrasound and use of a checklist during the scan were not significantly associated with missing images.

### 3.3 Image Quality

The 9849 images recorded were analysed by the expert (NF). The proportion of conformity criteria met in all images, and the scoring reliability between the operator and the expert, are detailed for all images in Table S2.

Of the 73 criteria selected for singleton pregnancies, 64 (88%) were met in over 90% of cases. Of the 9 criteria met in fewer than 90% of cases, 5 related to the recommended views: the visibility of the cavum septi pellucidi (85.4%), the visibility of at least one pulmonary vein to the left atrium (89.3%), the visibility of one adrenal gland on the AC view (63.7%), the visibility of the internal os (87.5%) and of the upper third of the cervix (87.2%) on a midsagittal view of the cervix. The 4 other criteria belonged to the additional views: the visibility of the right atrium (80.5%) and the visibility of the right ventricle (80.1%) on the short axis view of the heart, the visibility of the lateral ventricle (78.3%) and the visibility of the anterior part of the body of the corpus callosum (60.8%) on the cephalic coronal view.

### 3.4 Reliability Between Peer and Self‐Evaluation

All 88 participants analysed their own images. For 70/73 criteria (96%), the reliability between peer and self‐evaluation were good (AC1 > 0.80). No criterion had a poor reliability between the operator and the expert (AC1 < 0.6). Only 3 criteria had a moderate reliability between the operator and the expert (0.6 < AC1 < 0.8): the visibility of one adrenal gland on the AC view (AC1 = 0.67), the visibility of the ventricle on the short axis view of the heart (AC1 = 0.78) and the corpus callosum body on the cephalic coronal view (AC1 = 0.76).

### 3.5 Analysis of Additional Views

If the recommended images could not be obtained, 3 additional images (Views 22,23,24) could be produced as an alternative to illustrate the appearance of the abdominal wall with the umbilical insertion (View 16), the bifurcation of the pulmonary trunk (View 10) and the presence of the corpus callosum (View 3). However, the umbilical cord insertion was not deemed a conformity criterion by the experts for the recommended View 16.

If the pulmonary trunk bifurcation (Views 10) or the presence of the corpus callosum criteria were not met on the recommended images, additional views 23 and 24 were performed in 54% (19/35) and 50% (22/44) respectively, and the criteria was met by the expert in the additional view in 89% (17/19) and 36% (8/22) respectively.

### 3.6 Classification of Views Into Quality Groups

Among the 21 recommended views, 14 views were considered very highly compliant in the population (all conformity criteria were present at over 95% and an AC1 > 0.8), 3 views were considered highly compliant (at least one criterion between 90% and 95% and an AC1 > 0.8), and 4 views were considered moderately compliant (at least one criterion < 90% or an AC1 < 0.8). Among the 3 additional views, one was considered very highly compliant and 2 were considered moderately compliant (Figure S1).

## 4 Discussion

### 4.1 Main Findings

This large‐scale 2‐month ‘flash’ observational study demonstrates the feasibility and quality of a national standardised mid‐trimester ultrasound protocol [11].

Before assessing the feasibility of the views, we established a consensus‐based QA scoring system of 73 conformity criteria for 24 standardised views. This is essential, as it enables dissemination and teaching of these recommendations, external audits as part of continuous quality improvement programmes [14, 19, 20], provides objective criteria for retrospective analysis in cases of litigation, and paves the way for improved teaching of these standardised views.

To our knowledge, no other population‐based study assessed the feasibility of standardised views using pre‐established conformity criteria and consensus‐based QA scoring. Based on 9849 unselected images from consecutive scans in the general population, we observed feasibility ranging from 88.5% to 100%. Most conformity criteria (64/73, 88%) were met in over 90% of cases. In most cases, 70/73 (96%), Gwet's AC1 correlation between expert evaluation (peer‐evaluation) and participant evaluation (self‐evaluation) was greater than 0.80. This confirms the feasibility of quality image acquisition of the recommended views and defined quality criteria enabling objective and reproducible assessment of these views.

Finally, 19/24 (79%) of the recommended views were considered as highly or very highly compliant. Of the 4 views considered as moderately compliant, 3 were also those requiring the greatest number conformity criteria: the trans‐thalamic axial view, 4CV and AC view. It is well known that the more the criteria, the more difficult it is to meet all the criteria on the same image [21]. Furthermore, our study was performed by the full spectrum of professionals involved in routine foetal ultrasound with different level of expertise. The moderate agreement for several criteria may suggest that the screening operator may have false confidence in accuracy of the basic standard views, potentially marking them as correctly acquired when they are not, while failing to identify advanced views that are, in fact, correctly captured. This discrepancy could be explained by heterogeneous knowledge of the anatomic landmarks and their normality. Table S2 and Figure S1 illustrate the mapping of highly and moderately compliant views that enable us to establish focused teaching programmes to improve practice.

This audit also determined less important criteria such as the visibility of at least one adrenal gland on transverse view of the abdomen that is not routinely examined and reported in the mid‐trimester scan. These results suggest that the QA scoring should be revised and made more applicable in typical clinical practice.

Sonographers were encouraged to perform the 3 additional views. These views were obtained, and conformity criteria met, less often when they were imaged. Additional views were performed in 50% of cases when the recommended views were not performed, or when the structure to be identified was not visualised. This was the case for the visualisation of the corpus callosum. Moreover, the coronal view of the brain was of little use for visualising the corpus callosum in situations where it could not be examined on a midsagittal view, as it was not visible in approximately 2/3 of cases on the coronal view. This raises the question of the value of the concept of additional ‘screening’ views versus performing an advanced or targeted ultrasound examination and protocol when recommended conformity criteria cannot be met in routine screening studies.

### 4.2 Strengths and Weaknesses

Our study describes the essential steps involved in implementing a standardised ultrasound protocol on a national scale.

First is the definition of a protocol by experts, specifying the main views to be performed to optimise antenatal screening. These views should enable essential biometric and anatomical checks to be carried out verifying proper foetal development at the corresponding gestational age.

Second is to ensure the feasibility of these views in the population. This requires testing the views on a large scale, with widely representative operators and across various population groups, and also defining criteria for judging the quality of the views, similar to quality scores for biometry measurements [19].

The quality of the images, and the practicality of the quality criteria defined must then be confirmed, by checking their reliability and reproducibility between operators.

Tools are then available to improve quality, similar to the Deming wheel. The tools developed and presented in this article enable precise education of sonographers, self‐assessment of practice quality, peer‐assessment and, objective and constructive suggestions for quality improvement.

A main strength of this study is that ultrasound scans were performed by the full spectrum of professionals involved in routine foetal ultrasound, distributed across France, limiting potential selection bias. Membership in the diagnostic or screening group was not correlated with the number of recommended missing images (*p* = 0.35).

Selecting volunteer operators and a priori knowledge of the upcoming evaluation likely constitutes a bias [22]. However, asking for 5 consecutive scans limited this selection bias. The results of feasibility of additional images may imperfectly reflect their feasibility, as they were not truly considered mandatory. Peer‐evaluation by a single expert can also be considered a potential evaluation bias. However, use of a standardised consensus‐based scoring system helps reduce this bias. Although we observed good reliability between self‐evaluation and peer‐evaluation for most conformity criteria this QA scoring system, the results might have differed using a different system or by using different rules to evaluate agreement between reviewers. One might also decide to give different weights to different criteria in order to emphasise a particular aspect of quality. However, our results support the chosen criteria.

### 4.3 Interpretation

In 2022, ISUOG updated its mid‐trimester scan (between 18 and 24 weeks) guideline, proposing 21 views as a minimum standard [2]. The protocol used in our study is comparable, containing minimum standard views as well, plus three additional views: an axial abdominal view of the gallbladder, a para‐sagittal view of the left thoraco‐abdominal interface, and corpus callosum visualisation on a cephalic sagittal view. For each plane of the ISUOG protocol, the ISUOG guidelines detailed the anatomical structures that should be visualised, and for some of them quality criteria like image magnification.

The added value of the French QA scoring system is that each view has its own reading grid, comparable to the Herman score in the first trimester, enabling the operator not only to identify the structures to be checked, but also the expert assessing the quality of the image. As the quality of the images may reflect that of the US‐based foetal anatomical survey, poor image documentation precludes reinterpretation and could support allegations that an incomplete or inadequate US study has been performed. Our QA scoring system is consensus‐based and applicable in practice as we confirmed its reliability and reproducibility between operators. This enables self‐assessment of practice quality, peer‐assessment and, objective and constructive suggestions for quality improvement. To our knowledge no other recent guidelines, including the ISUOG guidelines, can be evaluated using such a process.

With quality standards and patient expectations increasing, greater medico‐legal and times pressures have been placed practitioners, requiring the documentation of high‐quality images and accurate reporting. Foetal ultrasound scores have been developed to improve standardisation of nuchal translucency measurement, as well as improving second‐ and third‐trimester ultrasound screening [19, 23, 24, 25].

Regular audit procedures are a means of improving and maintaining best practices [7, 26, 27]. Regular large‐scale external audits are time‐consuming and require highly qualified personnel. Consequently, self‐assessment of image acceptability by the operator is probably an effective element of training, and provides a good basis for constructive comparison with an external audit [28].

Recently, Yaqub et al. [26] have demonstrated the feasibility of improving completeness and quality of second‐trimester ultrasound examinations using a standardised protocol, by carrying out a clinical audit and a series of targeted actions over a one‐year period. In a first audit cycle, 103 501 ultrasound images from 6257 examinations performed; in a second cycle, 153 557 images from 6406 scans. The analysis was performed by including images obtained by the 20 sonographers who participated in both cycles. The authors observed an increase in the mean number of complete scans from 72% in the first year to 78% in the second evaluation (*p* < 0.001); an increase in median image quality score for all foetal views from 0.83 to 0.86 (*p* < 0.001). The improvement was greatest for sonographers with the lowest scores in the first audit.

Being able to complete protocols in an acceptable time frame and decreasing the need for additional exams affects broad applicability. In our study, 72.5% of exams were completed, with the more detailed examination (including 3 additional views) protocol, for an average examination time of 28 min. Longer examination time was significantly associated with the number of complete examinations (*p* = 0.016). However, an optimal threshold value could not be determined due to the poor diagnostic performance of this criterion (AUC = 0.516, 95% CI: 0.44–0.58). Cantazarite et al. had already demonstrated that a 30‐min examination time enabled a complete examination in 96% of cases after 20 weeks [29].

Other technical factors influencing the feasibility and quality of images in our study, such as BMI > 30 kg/m2 (*p* = 0.03), are concordant with the literature [30, 31]. Finally, the use of a software‐supported checklist, used by 21% of participants during the examination, was not correlated with higher completion rates (*p* = 0.13). Others report these tools improve the quantity and quality of images [32, 33], and would theoretically make examinations safer, similar to surgical checklists [34].

### 4.4 Conclusion

Results of this large population‐based study demonstrate the successful implementation of a recently developed national ultrasound protocol for the mid‐trimester scan.

This large‐scale audit study is essential, as it enables implementing an objective quality control and practice improvement approach based on self‐ and peer‐evaluation, which is essential for maintaining the quality of screening [35].

Training artificial intelligence (AI) tools using previously annotated images based on our consensual scoring system, may increase the efficiency of usually time and resource consuming large‐scale audits. As technology and AI advance, real‐time auditing could notify the operator of incomplete or low‐quality studies providing direct and immediate benefit to the quality of care delivered.

Development and adoption of standard imaging protocols, and importantly associated quality assessments tools, can reduce the inherent operator dependence of ultrasound and reduce ‘noise’ in obstetrical screening by introducing greater decision hygiene [36] and hopefully reducing errors. They can also, in the era of ‘value‐based care’, allow funders to proactively focus funding on higher quality pregnancy care and imaging by better aligning payment and measurable practice quality [37].

## Author Contributions

T.B.: planning, carrying out, analysing and writing. N.F.: planning, carrying out, analysing and writing. H.M.: writing. L.J.S.: planning and writing.

## Ethics Statement

This study was approved by the local ethical comity under CEROG 2023‐OBS‐0405.

## Conflicts of Interest

The authors declare no conflicts of interest.

## Supporting information


**Figure S1.** Recommended (solid border) and additional views (dashed border) as proposed by CNEOF 2022, represented as silhouettes classified into 3 population quality groups.

**Table S1.** Consensus‐based quality assessment scoring system for the 24 standardised views recommended by CNEOF 2022 for the mid‐trimester scan.

**Table S2.** Proportion of conformity criteria per view assessed by the expert and agreement between self‐evaluation and peer‐evaluation.

## Data Availability

The data that support the findings of this study are available from the corresponding author upon reasonable request. The data are not publicly available due to privacy or ethical restrictions.
