# Supplementary figures and images for: Feasibility of a Standardised Mid‐Trimester Ultrasound Protocol: A National Multicenter Study
Source: BJOG. 2025 Feb 13;132(8):1065–73. doi: 10.1111/1471-0528.18102 (PMC12137777; doi:10.1111/1471-0528.18102)

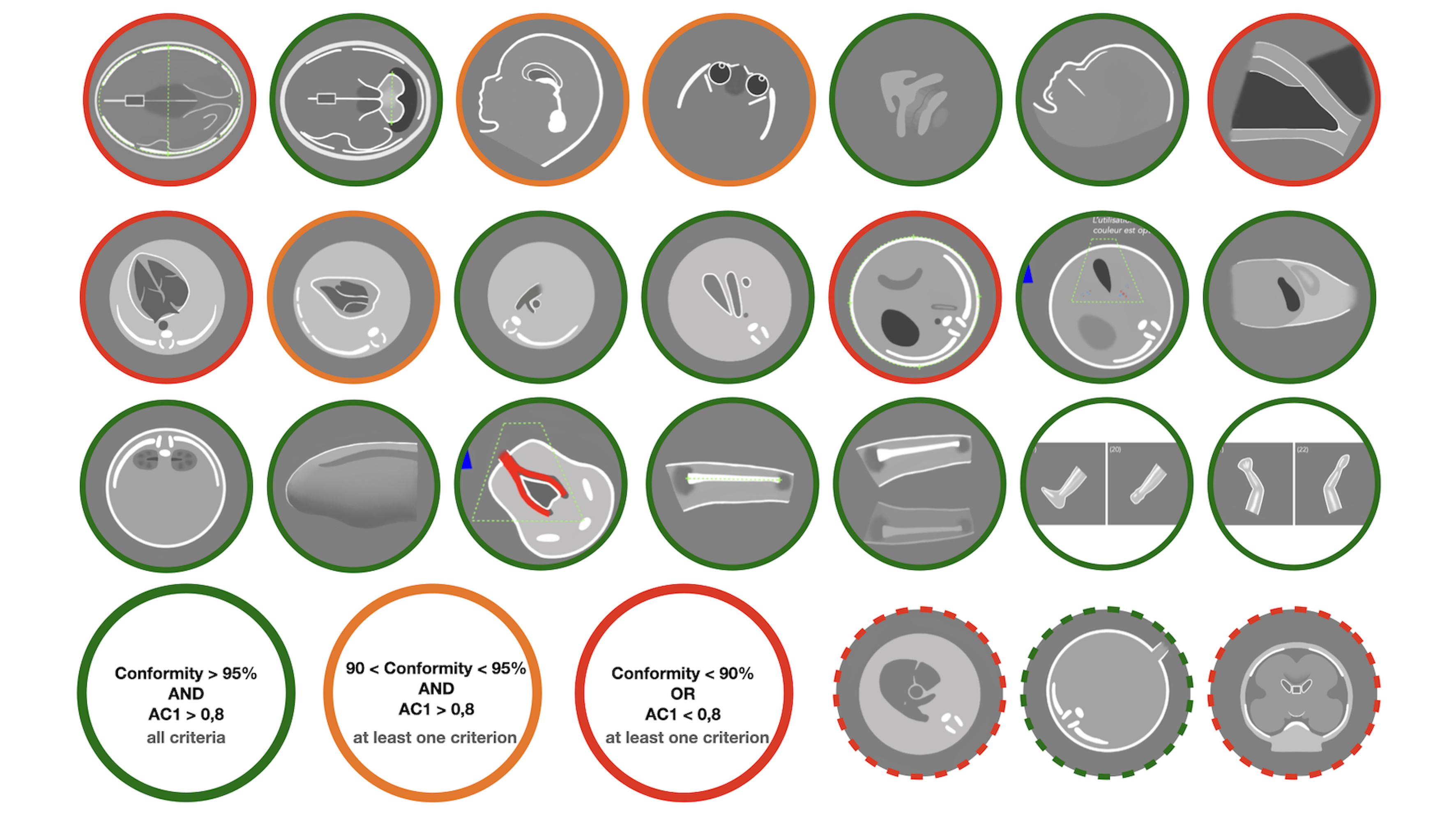

Supplement: Supplementary file 1 — Figure S1. Recommended (solid border) and additional views (dashed border) as proposed by CNEOF 2022, represented as silhouettes classified into 3 population quality groups. [file BJO-132-1065-s002.png]
