# Supplementary material for: Feasibility of a Standardised Mid‐Trimester Ultrasound Protocol: A National Multicenter Study
Source: BJOG. 2025 Feb 13;132(8):1065–73. doi: 10.1111/1471-0528.18102 (PMC12137777; doi:10.1111/1471-0528.18102)
Supplement: Supplementary file 2 — Table S1. Consensus‐based quality assessment scoring system for the 24 standardised views recommended by CNEOF 2022 for the mid‐trimester scan. [file BJO-132-1065-s001.docx]

| **QA scoring system for CNEOF recommended standardized Views** | **No. of Criteria** | **CNEOF silhouettes** |
| --- | --- | --- |
| **Image 1- Axial view of the cephalic pole** | | |
| The cavum of the septi pellucidi is visible | 7 | **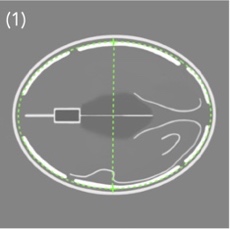** |
| The thalami are visible |  |  |
| The cerebellum is not visible |  |  |
| The midline is equidistant from the two sides of the skull |  |  |
| The calipers are positioned at the outer edges of the skull |  |  |
| The ellipse of the HC measurement is adjusted to the outer edge of the skull |  |  |
| The approach must be as perpendicular as possible |  |  |
| **Image 2- Trans-cerebellar axial oblique cephalic view** | | |
| Both cerebellar lobes are visible | 3 | 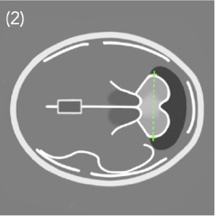 |
| The vermis is visible |  |  |
| The posterior cistern (cisterna magna) is visible |  |  |
| **Image 3- Midsagittal cephalic view** | | |
| The corpus callosum is visible | 1 | **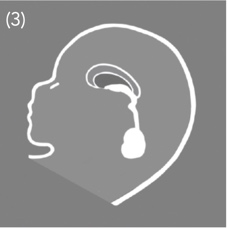** |
| **Image 4- Coronal view of the face centered on the nose and mouth** | | |
| Both nostrils are clearly identified | 3 | **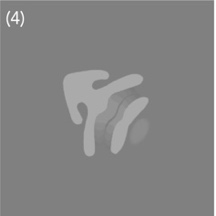** |
| The edge of the upper lip is visible |  |  |
| Continuity of the upper lip is visualized beyond the nostrils |  |  |
| **Image 5- Axial view of the orbits** | | |
| The two orbits are seen | 2 | 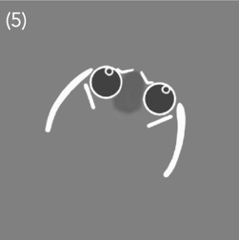 |
| The two crystalline lenses are seen |  |  |
| **Image 6- Midsagittal profile view** | | |
| Forehead clearly visible | 5 | 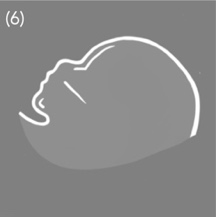 |
| Nose clearly visible |  |  |
| Lips clearly visible |  |  |
| Chin clearly visible |  |  |
| No orbit visible |  |  |
| **Image 7- Four-chamber view** | | |
| Crux of the heart is visible – AV valves crossing AV septum | 8 | **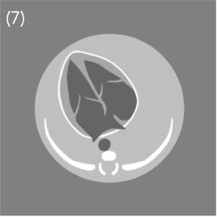** |
| Apex of the heart and 2 ventricles visible |  |  |
| Aorta seen behind the left atrium |  |  |
| The 4 chambers are visible |  |  |
| At least one pulmonary vein visualized at the left atrium |  |  |
| The interventricular septum is visible |  |  |
| The heart contours are clearly visible |  |  |
| The lungs are visible |  |  |

| **Image 8- Left outflow tract view** | | |
| --- | --- | --- |
| The interventricular septum is visible | 3 | **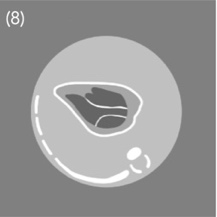** |
| Septo-aortic continuity is visible |  |  |
| The aorta is well followed beyond the aortic valve |  |  |
| **Image 9- Three vessels and trachea view** | | |
| Axial view of the thorax | 4 | 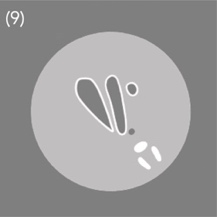 |
| The ductus arch is visible |  |  |
| The aorta is visible |  |  |
| The superior vena cava is visible |  |  |
| **Image 10- Bifurcation of the pulmonary artery view** | | |
| The bifurcation of the pulmonary artery (right pulmonary artery and ductus arteriosus) is visible | 1 | 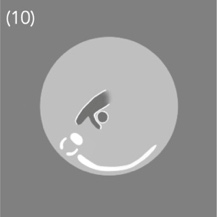 |
| **Image 11- Axial view of the abdomen through the portal sinus** | | |
| The portal sinus is visible | 6 | 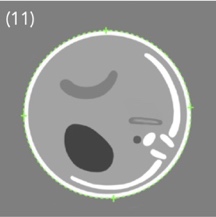 |
| The stomach is visible |  |  |
| At least one visible adrenal gland |  |  |
| The kidneys are not visible |  |  |
| The lungs are not visible |  |  |
| The ellipse of the abdominal perimeter measurement is adjusted and directly at the outer surface of the skin line |  |  |
| **Image 12- Axial view of the abdomen through the gallbladder** | | |
| The gallbladder is visible | 1 | 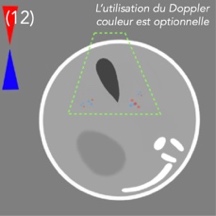 |
| **Image 13- Axial view of the kidneys** | | |
| The 2 renal pelvic cavities are visible | 2 | 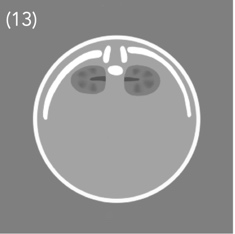 |
| The 2 kidneys are visible on either side of the spine |  |  |
| **Image 14- Midsagittal view of the lumbosacral spine** | | |
| The lumbosacral spine is visible | 2 | 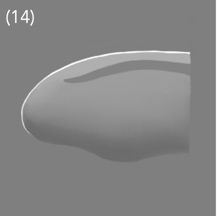 |
| The vertebral bodies are visible |  |  |

| **Image 15- Left parasagittal view of thorax and abdomen** | | |
| --- | --- | --- |
| The stomach is visible | 2 | 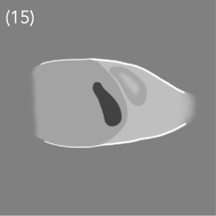 |
| The entire length of the left diaphragmatic interface is visible |  |  |
| **Image 16- Oblique view of the pelvis through the umbilical arteries and the bladder** | | |
| The bladder is visible | 2 | 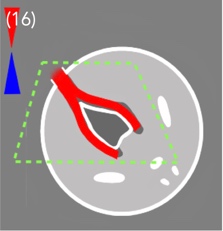 |
| The umbilical arteries are visible on either side of the bladder |  |  |
| **Image 17- Longitudinal view of the femur** | | |
| The femur is horizontal (or less than 20°) | 5 | 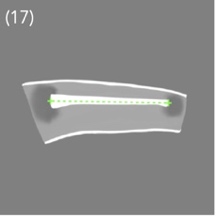 |
| Each caliper is placed at the ends of the ossified diaphysis |  |  |
| The diaphysis is straight |  |  |
| Ends are sharp and perpendicular to the ultrasound beam |  |  |
| The femur measured is closest to the probe |  |  |
| **Image 18- Cross-view of the 2 femurs** | | |
| The 2 femurs are visible on 1 image or 2 images at the same scale | 1 | 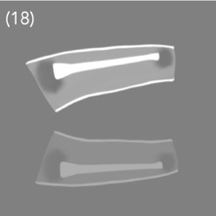 |
| **Image 19- Lower limbs view** | | |
| Legs and hindfeet are visible on 2 images in sagittal or coronal view | 1 | 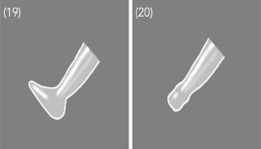 |
| **Image 20- Upper limbs view** | | |
| The forearms and hands are visible in 2 images | 1 | 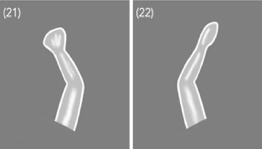 |
| **Image 21- Midsagittal view of the uterus through the cervix** | | |
| Internal os is visible | 3 | **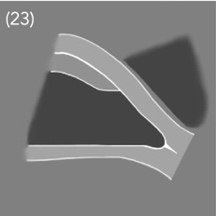** |
| The upper 1/3 of the cervix is visible |  |  |
| The image is centered on the internal os of the cervix |  |  |

| **QA scoring system for CNEOF additional standardized Views** | **No of Criteria** | **CNEOF silhouettes** |
| --- | --- | --- |
| **Image 22- Axial view of the abdomen at insertion level** | | |
| Umbilical cord insertion is visible | 1 | 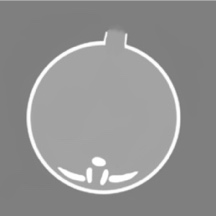 |
| **Image 23- View of the right sided cardiac cavities surrounding the aorta** | | |
| The right atrium is visible | 1 | **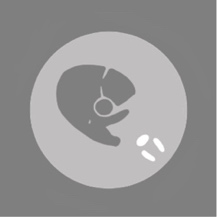** |
| The right ventricle is visible |  |  |
| The pulmonary bifurcation is visible |  |  |
| The aorta is visible at the center of the looped right sided structures |  |  |
| **Image 24- Coronal view of the cephalic pole** | | |
| The cavum of the septi pellucidi is visible | 5 | **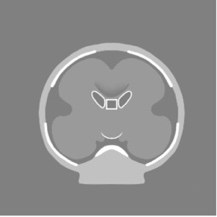** |
| The lateral ventricles are visible |  |  |
| The inter-hemispheric fissure is visible |  |  |
| The anterior part of the body of the corpus callosum is visible |  |  |
| Hemispheres are symmetrical |  |  |
