# Supplementary material for: Feasibility of a Standardised Mid‐Trimester Ultrasound Protocol: A National Multicenter Study
Source: BJOG. 2025 Feb 13;132(8):1065–73. doi: 10.1111/1471-0528.18102 (PMC12137777; doi:10.1111/1471-0528.18102)
Supplement: Supplementary file 3 — Table S2. Proportion of conformity criteria per view assessed by the expert and agreement between self‐evaluation and peer‐evaluation. [file BJO-132-1065-s003.docx]

Table S2. Proportion of conformity criteria per view assessed by the expert and agreement between self-evaluation and peer-evaluation

| **Criterion** | **Presence**  **of conformity criterion**  **Operator**  **(%)** | **Presence**  **of conformity criterion**  **Expert**  **(%)** | **Concordance**  **Self and peer-assessment**  **(AC1 from Gwet)** |
| --- | --- | --- | --- |
| **Recommended standardized view** | | | |
| **Axial view of the cephalic pole** | | | |
| **The cavum of the septi pellucidi is visible** | 92.2 | **85.4** | 0.87 |
| **The thalami are visible** | 94.8 | **91.3** | 0.91 |
| The cerebellum is not visible | 96.1 | 97.7 | 0.96 |
| The midline is equidistant from the two sides of the skull | 94.8 | 99.3 | 0.95 |
| The calipers are positioned at the outer edges of the skull | 96.3 | 98.6 | 0.96 |
| The ellipse of the head circumference measurement is adjusted to the outer edge of the skull | 96.1 | 98.6 | 0.96 |
| The approach must be as perpendicular as possible | 96.6 | 98.9 | 0.96 |
| **Trans-cerebellar axial oblique cephalic view** | | | |
| Both cerebellar lobes are visible | 99.5 | 99.5 | 0.99 |
| The vermis is visible | 95.2 | 98.4 | 0.94 |
| The posterior cistern/cisterna magna is visible | 97.0 | 99.3 | 0.97 |
| **Midsagittal cephalic view** | | | |
| **The corpus callosum is visible** | 92.2 | **94.6** | 0.92 |
| **Coronal view of the face centered on the nose and mouth** | | | |
| Both nostrils are clearly identified | 97.9 | 99.5 | 0.97 |
| The edge of the upper lip is visible | 99.1 | 99.8 | 0.98 |
| Continuity of the upper lip is visualized beyond the nostrils | 97.0 | 99.8 | 0.96 |
| **Axial view of the orbits** | | | |
| The two orbits are seen | 98.4 | 99.1 | 0.98 |
| **The two crystalline lenses are seen** | 87.9 | **90.5** | 0.88 |
| **Midsagittal profile view** | | | |
| Forehead clearly visible | 98.4 | 1 | 0.98 |
| Nose clearly visible | 99.1 | 99.8 | 0.99 |
| Lips clearly visible | 97.9 | 99.1 | 0.99 |
| Chin clearly visible | 95.4 | 97.0 | 0.96 |
| No orbit visible | 97.7 | 99.3 | 0.97 |
| **Four-chamber view** | | | |
| **Crux of the heart is visible – AV valves crossing AV septum** | 95.2 | **91.8** | 0.89 |
| Apex of the heart and 2 ventricles visible | 92.9 | 99.8 | 0.93 |
| **Aorta seen behind the left atrium** | 95.9 | **94.5** | 0.95 |
| The 4 chambers are visible | 99.3 | 1 | 0.99 |
| **At least one pulmonary vein visualized at the left atrium** | 93.6 | **89.3** | 0.88 |
| The interventricular septum is visible | 96.6 | 98.6 | 0.96 |
| The heart contours are clearly visible | 92.2 | 1 | 0.92 |
| The lungs are visible | 96.3 | 99.8 | 0.96 |
| **Left outflow tract view** | | | |
| **The interventricular septum is visible** | 94.0 | **94.7** | 0.90 |
| Septo-aortic continuity is visible | 96.1 | 96.3 | 0.93 |
| The aorta is well followed beyond the aortic valve | 96.7 | 95.2 | 0.94 |
| **Three vessels and trachea view** | | | |
| Axial view of the thorax | 91.2 | 97.7 | 0.91 |
| The ductus arch is visible | 95.4 | 97.9 | 0.93 |
| The Aorta is visible | 96.8 | 97.2 | 0.95 |
| The superior vena cava is visible | 95.8 | 96.5 | 0.94 |
| **Bifurcation of the pulmonary artery view** | | | |
| The bifurcation of the pulmonary artery (right pulmonary artery and ductus arteriosus) is visible | 96.7 | 95.2 | 0.96 |
| **Axial view of the abdomen through the portal sinus** | | | |
| The portal sinus is visible | 92.7 | 95.0 | 0.91 |
| The stomach is visible | 99.3 | 96.8 | 0.97 |
| **At least one visible adrenal gland** | 89.9 | **63.7** | **0.67** |
| **The kidneys are not visible** | 97.7 | **90.9** | 0.91 |
| The lungs are not visible | 97.3 | 95.9 | 0.94 |
| The ellipse of the abdominal perimeter measurement is adjusted and directly at the outer surface of the skin line | 94.5 | 98.6 | 0.94 |
| **Axial view of the abdomen through the gallbladder** | | | |
| The gallbladder is visible | 99.1 | 99.1 | 0.98 |
| **Axial view of the kidneys** | | | |
| The 2 renal pelvic cavities are visible | 92.6 | 96.8 | 0.91 |
| The 2 kidneys are visible on either side of the spine | 95.1 | 98.8 | 0.96 |
| **Midsagittal view of the lumbosacral spine** | | | |
| The lumbosacral spine is visible | 93.3 | 96.7 | 0.92 |
| The vertebral bodies are visible | 92.6 | 99.5 | 0.97 |
| **Left parasagittal view of thorax and abdomen** | | | |
| The stomach is visible | 97.5 | 96.8 | 0.97 |
| The entire length of the left diaphragmatic interface is visible | 95.4 | 95.4 | 0.95 |
| **Oblique view of the pelvis through the umbilical arteries and the bladder** | | | |
| The bladder is visible | 99.0 | 98.6 | 0.99 |
| The umbilical arteries are visible on either side of the bladder | 92.6 | 99.3 | 0.93 |
| **Longitudinal view of the femur** | | | |
| The femur is horizontal (or less than 20°) | 93.4 | 99.5 | 0.93 |
| Each caliper is placed at the ends of the ossified diaphysis | 98.6 | 98.6 | 0.98 |
| The diaphysis is straight | 97.7 | 1 | 0.98 |
| Ends are sharp and perpendicular to the ultrasound beam | 97.3 | 1 | 0.97 |
| The femur measured is closest to the probe | 99.5 | 1 | 0.99 |
| **Cross-view of the 2 femurs** | | | |
| The 2 femurs are visible on 1 image or 2 images at the same scale | 93.8 | 98.4 | 0.94 |
| **Lower limbs view** | | | |
| Legs and hindfeet are visible on 2 images in sagittal or coronal view | 92.1 | 99.0 | 0.92 |
| **Upper limbs view** | | | |
| The forearms and hands are visible in 2 images | 82.3 | 98.3 | 0.82 |
| **Midsagittal view of the uterus through the cervix** | | | |
| **Internal os is visible** | 85.6 | **87.5** | 0.88 |
| **The upper 1/3 of the cervix is visible** | 79.5 | **87.2** | 0.83 |
| **The image is centered on the internal os of the cervix** | 94.1 | **92.2** | 0.90 |
| **Additional standardized views** | | | |
| **Transverse view of the abdomen at insertion level** | | | |
| Umbilical cord insertion is visible | 95.9 | 99.1 | 0.96 |
| **Cross-view of the right cavities curling up around the aorta** | | | |
| **The right atrium is visible** | 83.7 | **80.5** | 0.82 |
| **The right ventricle is visible** | 83.7 | **80.1** | **0.78** |
| **The pulmonary bifurcation is visible** | 97.6 | **92.8** | 0.94 |
| The aorta is visible at the center of the looped right sided structures | 98.0 | 97.6 | 0.97 |
| **Coronal view of the cephalic pole** | | | |
| **The cavum of the septi pellucidi is visible** | 94.0 | **90.2** | 0.92 |
| **The lateral ventricles are visible** | 81.7 | **78.3** | 0.83 |
| The inter-hemispheric fissure is visible | 91.5 | 95.3 | 0.91 |
| **The anterior part of the body of the corpus callosum is visible** | 76.6 | **60.8** | **0.76** |
| Hemispheres are symmetrical | 88.9 | 97.9 | 0.88 |

*Compliance criteria are bolded when the percentage of compliance criteria validation by expert is < 95% or when AC1< 0.8.*

*In green, views considered to be very well compliant (all compliance criteria present at over 95% and AC1 > 0.8).*

*In orange, views considered to be well compliant (>1 compliance criteria present between 90 and 95% and AC1 > 0.8).*

*In red, views considered as moderately compliant (>1 compliance criteria present at less than 90% or an AC1 < 0.8).*
